# Acquired distance esotropia associated with myopia in the young adult

**DOI:** 10.1186/s12886-018-0717-2

**Published:** 2018-02-20

**Authors:** Ke Zheng, Tian Han, Yinan Han, Xiaomei Qu

**Affiliations:** Department of Ophthalmology, Eye and ENT Hospital of Fudan University and Myopia Key Laboratory of Ministry of Health, Shanghai, China

**Keywords:** Distance esotropia, Diplopia, Myopia

## Abstract

**Background:**

To describe the clinical features of acquired progressive esotropia, with a larger angle at distance than near, associated with myopia in young adults.

**Methods:**

Eleven adults (ages ranging from 18 to 37 years) with constant or intermittent horizontal diplopia at distance were recruited. Subjective refraction, ocular alignment, fusional amplitudes and horizontal eye movements were measured at distance and near.

**Results:**

Distance esotropia varied from 20 to 60 prism diopters (PD). At near, the esotropic deviation ranged from 10 to 30 PD. Spherical equivalents (SE) of the right eye ranged from − 3.50 to − 8.25 diopters (D) while SE of the left eye ranged from − 0.375 to − 7.25 D. Ten of the eleven patients presented with constant diplopia at distance. Horizontal ductions and versions were full in all patients. The pathological report of seven patients who underwent lateral rectus resection showed that there were no muscle fibres, but rather, collagenous fibres.

**Conclusions:**

This unusual sub-type of strabismus is a benign entity with slow progression that can occur in young adults with myopia. The cause of this condition is still unknown, and may be related to long periods of near work.

## Background

Comitant esotropia is generally divided into three main categories: infantile-onset esotropia, accommodative esotropia, and non-accommodative acquired esotropia. In a previous report, age-related distance esotropia was proposed as a new subcategory. David Mittelman [1] reported this kind of non-accommodative acquired esotropia occurring in older patients with a median age of 77 years. Daisy Godts [2] reported this form of esotropia as being observed in patients over the age of 60, and this form was not associated with any neurological abnormalities or lateral rectus underaction. Divergence insufficiency (DI) presented comitant esotropia that is greater at distance than at near due to the progressive loss of fusional divergence amplitudes [3]. The patients usually were without neurological abnormalities and presented with the insidious onset of horizontal diplopia at distance.

In our clinical practice, the young adults who have acquired distance esotropia have some similarities with the signs of acquired progressive esotropia, typically a larger angle at distance than near and they tend to present with an insidious onset of horizontal diplopia at distance. This is usually associated with prolonged near work and myopia. By describing the clinical characteristics of these patients, hopefully it can be understood whether these patients belong to a special type of esotropia, and the possible pathogenesis can be explored.

## Subjects

Eleven adult patients with myopia and distance esotropia were recruited and assessed between January 2015 and February 2015. The inclusion criteria included normal ductions, onset of symptoms before the age of 40, comitancy of esotropia in lateral gazes, symptoms of diplopia at distance and an esotropia greater at distance than near by five prism diopters (PD) or more. The patients who had a history of orbital trauma, childhood strabismus, previous strabismus surgery, accommodative disorders, coexisting vertical strabismus, myasthenia gravis, thyroid eye disease, cranial nerve palsies or supranuclear palsies would be excluded.

All patients had corrected visual acuities of 20/20 or better. All were evaluated using the duochrome test to ensure that they were not overcorrected. Vergence fusional amplitudes were measured with a phoropter. Binocular alignment was measured using the prism cover test. Ocular motility was evaluated clinically in all directions of gaze with an emphasis on lateral gaze. A portion of the lateral rectus muscle was taken for Haematoxylin-Eosin staining in patients who underwent lateral rectus resection.

This study followed the tenets of the Declaration of Helsinki and was approved by the ethics committee of the Eye and ENT Hospital of Fudan University. Informed consent was obtained from all participants.

## Results

Eleven young adults were selected for this study: five females and six males. The patients ranged in age from 18 to 37 years, with a median age of 25 years. Table 1 shows that the spherical equivalents (SE) of the right eye ranged from − 3.50 to − 8.25 diopters (D), and the mean SE was − 5.65 DS; the SE of the left eye ranged from − 0.375 to − 7.25 D, and the mean SE was − 5.09 D. All patients complained of horizontal diplopia on distance fixation. The duration of these symptoms ranged from three months to seven years, with a mean of 32 months. Constant diplopia was the presenting complaint in 10 of the 11 individuals. All the patients had a history of prolonged near work, ranging from 6 h to 13 h per day (a median time of 12 h per day), before the onset of diplopia. Table 2 shows that the distance deviation varied from 20 PD esotropia to 60 PD esotropia, with a median angle of 30 PD esotropia. At near fixation, the measurements ranged from 10 PD esotropia to 30 PD esotropia, with a median deviation of 20 PD esotropia. Ductions and versions were full. There was no evidence of lateral rectus paresis. The deviation difference was less than 5 PD in various positions of gaze. None of the patients had an obvious underlying neurological disease such as tumour or stroke. Three patients had previously undergone computed axial tomography (CT) or magnetic resonance imaging (MRI) after the onset of diplopia, the results of which were found to be normal for their age. None of the patients displayed a vertical deviation.

**Table 1:** Patient characteristics

Case No.	SE of OD(D)	SE of OS(D)	Medical History	Head Scan	prolonged near work time(h)	Onset (months)
1	−7	−7.25	–	–	6	84
2	−5.5	−5.25	–	–	8	3
3	−5.5	−5.125	–	CT/MRI:normal	6	84
4	−8.25	−6.875	–	CT:normal	13	60
5	−5	−5.25	–	–	10	24
6	−6.875	−6.75	–	–	12	24
7	−3.875	−0.375	–	–	12	24
8	−3.5	−3.75	–	–	8	12
9	−6.725	−5.5	–	–	12	8
10	−4.75	−5	–	MRI:normal	12	5
11	−5.25	−4.875	–	–	13	24

*SE* Spherical equivalents, *D* Diopter

**Table 2:** Deviation and fusional amplitudes at distance and near

Case No.	Distance Deviation(PD)	Near Deviation(PD)	Distance BI(PD)	Distance BO(PD)	Near BI(PD)	Near BO(PD)
1	20	10	5.2/21.8/18.8	5.8/7.4/5	x/3.2/1.6	x/3.4/2.8
2	40	30	diplopia	diplopia	diplopia	diplopia
3	60	10	diplopia	diplopia	3/7.2/6	25/> 40/26
4	30	20	diplopia	diplopia	diplopia	diplopia
5	30	20	diplopia	diplopia	diplopia	diplopia
6	30	25	diplopia	diplopia	5.8/8/0.8	11/> 40/12
7	40	20	diplopia	diplopia	x/3.2/0.4	20/34.8/28.2
8	30	20	diplopia	diplopia	diplopia	diplopia
9	40	30	diplopia	diplopia	diplopia	diplopia
10	20	10	diplopia	diplopia	5.2/5.8/1	28/> 40/30
11	35	30	diplopia	diplopia	diplopia	diplopia

**Fig. 1:**
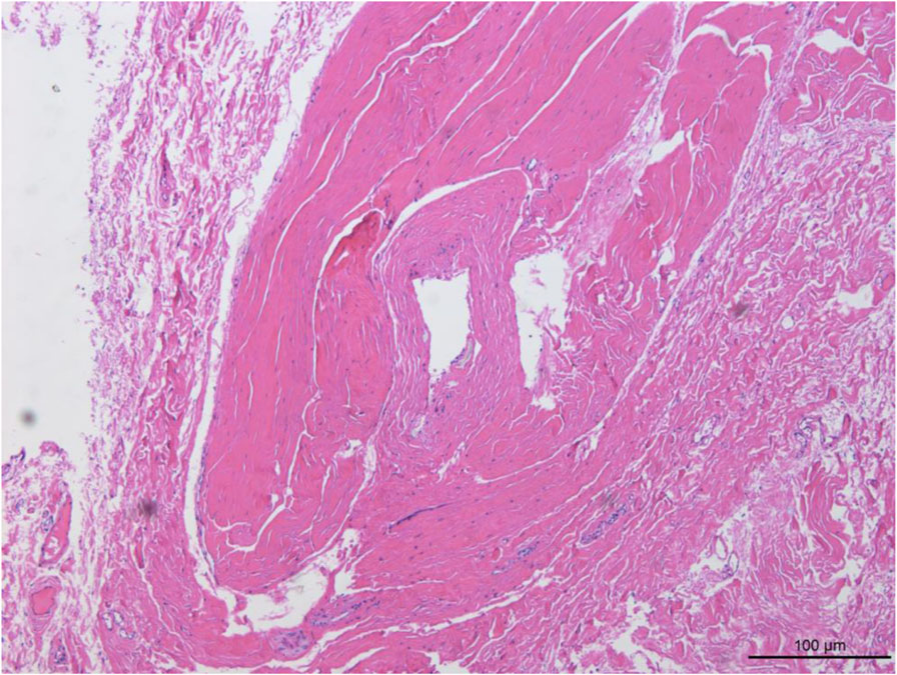
Lateral rectus histopathology of patient 5

*PD* Prism diopters, *BI*:Base in, *BO* Base out;

Seven patients were successfully treated with lateral rectus resection, and all samples underwent pathological examination, which showed that there were no muscle fibres but rather collagenous fibres from the lateral rectus. Figure 1.

## Discussion

A previous population-based study reported that distance esotropia with orthotropia at near may include as many as 10.6% of adults with strabismus [4]. The present study is the first to report the features of distance esotropia in young adults. However, the classification of esotropia at distance has not been entirely clear because many clinical situations may produce an esotropia greater at distance than near. Divergence palsy affects all age groups and is often associated with various neurological disorders, and the sudden onset of distance esotropia is associated with loss of divergence [1]. None of the patients in the presented study had any neurological pathology.

Medial rectus muscle restriction with Graves’ disease may cause comitant esotropia at distance [5]; however, none of the patients in the present study showed any ocular or systemic symptoms or signs of Graves’ disease. Orbital lipoatrophy can reduce muscle tension in extraocular muscles by a relative decrease in muscle strain. The stronger medial rectus muscle, which is associated with relative relaxation of the lateral rectus muscle, may result in a distant esotropia [6]. The finding is often associated with a relative and/or progressive enophthalmos, which was not seen in the present study group, making this aetiology less likely. Recently, Rutar and Demer [7] showed that orbital connective tissue degeneration led to a sudden decline in three elderly patients. However, the patients in the present study are young adults.

The aetiology of this uncommon type of strabismus is unknown by the authors, but there is a tendency to speculate that progressive myopathy affects the lateral rectus. The elderly may appear to have similar limitations. Bothun and Archer [5] reported a series of eight older patients (mean age of 60 years) with progressive distance esotropia; all patients were healthy and didn’t have any neurological diseases. Robert E. Wiggins [8], in his series of elderly patients (mean age of 72 years), defined the divergence weakness as comitant esotropia with diplopia at distance and fusion at near. Horizontal ductions and versions should be normal. Christine Berscheid [9] showed that patients with secondary DI have the tendency to present with diplopia at a younger age than those with primary DI. The average age of patients with DI in the study was 51 years, compared with 62 years in patients with primary DI. It is suggested that the distance esotropia was possibly due to vascular factors in elderly patients. In the present study, the mean patient age is 25 years, which is much younger than the ages in previous studies.

Guyton [10] has proposed that tight adduction from near-target convergence leads to a shortening of the medial rectus muscle and reduced ability to maintain orthographic position at distance. In the present study, it was found that all patients spent a prolonged period performing tasks requiring near vision, ranging from six hours to 13 h per day (a median time of 12 h per day), before the onset of double vision. Therefore, it is hypothesized that the esotropia described in this article is due to the weakening of the lateral rectus muscle from prolonged near work, resulting in a slow progressive esodeviation that manifests initially at distance. Seven patients underwent a pathological examination of their lateral rectus muscles, which showed that there were no muscle fibres but rather collagenous fibres. Small esodeviations can be easily corrected because divergent fusion is often prioritized. However, divergent fusional amplitudes are much smaller at distance. Therefore, when the eyes deviate progressively inwards, patients are unable to compensate, resulting in horizontal diplopia at distance. Akiko Tanaka [11] reported that exotropia was more common than esotropia in patients with pathologic myopia. High myopia prescriptions are often undercorrected for high refractive error, which would induce less accommodation and exotropia. However, in the present study, all patients were myopic and had distance esotropia. Clearly, many more patients need to be studied in the future.

## Conclusions

In conclusion, acquired distance esotropia with diplopia was observed in young adults with myopia; however, the aetiology is still unclear. It can be hypothesized that the esotropia described is due to the weakening of the lateral rectus muscle from prolonged near work, resulting in a slow, progressive esodeviation that manifests initially at distance.

## Abbreviations

BI: Base in
BO: Base out
CT: Computed axial tomography
D: Diopters
DI: Divergence insufficiency
MRI: Magnetic resonance imaging
PD: Prism diopter
SE: Spherical equivalents
